# Applications of Replicating-Competent Reporter-Expressing Viruses in Diagnostic and Molecular Virology

**DOI:** 10.3390/v8050127

**Published:** 2016-05-06

**Authors:** Yongfeng Li, Lian-Feng Li, Shaoxiong Yu, Xiao Wang, Lingkai Zhang, Jiahui Yu, Libao Xie, Weike Li, Razim Ali, Hua-Ji Qiu

**Affiliations:** 1 State Key Laboratory of Veterinary Biotechnology, Harbin Veterinary Research Institute, Chinese Academy of Agricultural Sciences, 427 Maduan Street, Harbin 150001, China; 15204662904@126.com (Y.L.); lilianfeng124603@163.com (L.-F.L.); yushaoxiong1987@hotmail.com (S.Y.); wangxiao2037@126.com (X.W.); tdzlk1991@163.com (L.Z.); yujiahui2014@126.com (J.Y.); xielibao2015@163.com (L.X.); 2 Department of Chemistry, College of Arts and Sciences, Georgia State University, Atlanta, GA 30302, USA; wli22@gsu.edu; 3 University of Karachi, Karachi 75270, Pakistan; razimalikhan@gmail.com

**Keywords:** replicating-competent virus, reporter, high-throughput screening, molecular virology

## Abstract

Commonly used tests based on wild-type viruses, such as immunostaining, cannot meet the demands for rapid detection of viral replication, high-throughput screening for antivirals, as well as for tracking viral proteins or virus transport in real time. Notably, the development of replicating-competent reporter-expressing viruses (RCREVs) has provided an excellent option to detect directly viral replication without the use of secondary labeling, which represents a significant advance in virology. This article reviews the applications of RCREVs in diagnostic and molecular virology, including rapid neutralization tests, high-throughput screening systems, identification of viral receptors and virus-host interactions, dynamics of viral infections *in vitro* and *in vivo*, vaccination approaches and others. However, there remain various challenges associated with RCREVs, including pathogenicity alterations due to the insertion of a reporter gene, instability or loss of the reporter gene expression, or attenuation of reporter signals *in vivo*. Despite all these limitations, RCREVs have become powerful tools for both basic and applied virology with the development of new technologies for generating RCREVs, the inventions of novel reporters and the better understanding of regulation of viral replication.

## 1. Introduction

The commonly used tests based on wild-type viruses, such as immunostaining, are often time-consuming and labor-intensive. Furthermore, these methods cannot meet the demands for high-throughput screening (HTS) of antivirals, rapid, sensitive and quantitative detection of neutralizing antibodies (NAbs), visual tracking of viral proteins or viruses *in vitro* and *in vivo* and other fields of virology.

Replicating-competent reporter-expressing viruses (RCREVs) are one type of artificially modified viruses that not only retain the viral genetic characteristics but also possess the new properties of the reporter genes, which represent a useful tool for quantitative analysis of viral replication and tracking viral protein transport in both living cells and animals.

## 2. Technologies for the Generation of Replicating-Competent Reporter-Expressing Viruses (RCREVs)

To date, advances in technologies enable the generation of RCREVs, which have been successfully applied in diagnostic and molecular virology.

### 2.1. Reverse Genetics Technologies

Currently, reverse genetics systems for many viruses have been well-established [1,2,3,4,5,6,7,8,9,10,11,12,13], providing powerful tools for generating RCREVs. Since some viruses possess a large genome, they usually permit a large extrinsic genetic insertion without impairing viral replication. For example, vaccinia virus (VACV) contains a 192 kb genome, capable of accepting up to 25 kb insertion [14]. However, for most RNA and some DNA viruses containing a small-sized genome, a recurring difficulty in generating RCREVs is the genetic instability, especially for a larger reporter gene. For some viruses with a segmented RNA genome, the insertion of a large reporter gene into the genome is difficult or even impossible to achieve.

### 2.2. Reporters in RCREVs

Commonly used reporters in RCREVs include fluorescent proteins, such as enhanced green fluorescent protein (EGFP) (green), GFP mutants (enhanced cyan fluorescent protein (ECFP) (blue), mCherry (red) and Venus (yellow)), far-red fluorescent reporters (red fluorescent protein (RFP), Katushka 2, dTomato and DsRed)), near-infrared fluorescent proteins (iRFPs) and tetracysteine (TC); bioluminescent reporters, such as firefly luciferase (Fluc), *Renilla* luciferase (Rluc) and Gaussia luciferase (Gluc); in addition to other reporters, such as neomycin-resistance gene (Neo^R^) and Cre recombinase. These reporters are mainly used to rapidly quantify viral replication and track viral proteins or viruses by living imaging *in vitro* and *in vivo*. However, different reporters may have different influences on the biological properties of various viruses, and the loss of the reporter gene expression is a significant concern for some RCREVs. Therefore, choosing a suitable reporter is a critical decision on designing RCREVs. For the planned applications, the reporters with a smaller size may be a promising option due to their minimum effects on the viral biology. For example, the Rluc gene (933 bp) is better than the Fluc gene (1653 bp) and has a minimal influence on the growth of the engineered classical swine fever virus (CSFV) expressing the reporters [15,16].

### 2.3. Reporters Expressing Strategies

Various strategies associated with the reporter gene expression have been developed. An extensively used expression strategy is to fuse the reporters to one of the viral proteins. For instance, the Rluc activities from engineered CSFV carrying the Rluc fused to the viral N^pro^ protein were detected [15]. A nonessential viral gene can be replaced with a reporter gene to generate a reporter virus. In addition, the Cre-LoxP recombination is widely used to control reporter gene expression in cell cultures or animal models. Notably, reporters can be expressed from an additional transcriptional unit (ATU), in which a reporter gene is generally flanked by highly conserved gene start-and-stop signals. For instance, GFP was expressed as a separate protein from the ATU in the recombinant peste des petits ruminants virus (PPRV) [17]. Furthermore, the reporters can be expressed separately by introduction of an internal ribosome entry site (IRES) or foot-and-mouth disease virus (FMDV) 2A self-cleaving peptide (2A) (LLNFDLLKLAGDVESNPG↓P), which is able to undergo self-cleavage allowing simultaneous expression. For example, recombinant alphaviruses expressing a separate Fluc by 2A-mediated cleavage were successfully used to screen viral receptors [18].

Since the properties of RCREVs and the stability of reporter genes may vary among different strategies, the selection of expression strategy is another principal consideration on designing RCREVs for specific applications. Notably, the same strategy might lead to different effects on the growth of the same virus due to the distinct insertion site. For example, a recombinant respiratory syncytial virus (RSV) expressing a reporter protein from an ATU upstream of NS1 displayed negligible attenuation in cell cultures [19], whereas the RSV expressing a reporter from an ATU inserted between F and G genes was significantly attenuated in cell cultures [20]. Additionally, the use of 2A peptide to achieve expression of a separate reporter might constitute a promising approach as 2A peptide is small and can readily be self-cleaving while minimizing the possibility of the loss of functions of the viral proteins.

## 3. Applications of RCREVs in Serum-Virus Neutralization Tests

The neutralization immunofluorescence test (NIFT) is currently a gold standard for the detection of NAbs against many noncytopathogenic viruses. However, NIFT is labor-intensive and time-consuming due to the necessary incubation and staining procedures. It would be convenient to use RCREVs to detect NAbs directly without immunostaining. There are many successful applications of RCREVs harboring EGFP, Rluc or Fluc in the rapid neutralization tests [17,21,22,23,24,25].

For viruses causing slight or no cytopathic effects (CPEs) in cultured cells, the EGFP reporter can be chosen to generate RCREVs for quantifying NAbs with higher specificity through direct observation of EGFP fluorescence. Due to the structural characteristics of EGFP, the fluorescence of EGFP fused to a viral protein may be attenuated or quenched. Therefore, EGFP should be separated from the viral protein by introduction of an ATU, IRES or 2A sequence when constructing the RCREVs. Owing to the simple assaying for Gluc activity compared with the Fluc, Rluc and other bioluminescent reporters, it is advantageous to determine the neutralizing antibody titers based on Gluc-expressing viruses.

Notably, attenuated RCREVs can also be applied for rapid neutralization tests due to high sensitivity and operational simplicity for detection of the reporters.

## 4. Application of RCREVs in Screening Systems

Antiviral compounds, interferon-stimulated genes (ISGs) or small interfering RNAs (siRNAs) have potential applications in the treatment of many diseases. The traditional screening methods of them are developed by a cell-based HTS, in which the treated cells were observed under a microscope for the inhibitory activity of the compounds for CPEs [26], enzyme-linked immunosorbent assay (ELISA) [27] or fluoresces-linked immunosorbent assay [28]. Using these approaches, the scientists have screened and identified a series of small antiviral molecules or inhibitors [29,30]. However, the traditional methods based on wild-type viruses are inefficient for antiviral screening.

To overcome this problem, RCREVs have been applied for the purpose of antiviral screening, because RCREVs can target the complete virus life cycle and offer a higher throughput of antiviral screening than traditional assays. RCREVs represent powerful screening tools for identifying antiviral compounds against various highly pathogenic viruses [31,32,33,34]. For example, a high-throughput assay for Zaire EBOV has been established using the recombinant EBOV expressing the EGFP reporter gene [31]. Interestingly, reporter viruses in combination with other approaches, such as RNAi, have been applied to screen anti-CSFV ISGs [15], which is time- and cost-effective. Importantly, RCREVs with slightly reduced growth ability compared with the wild-type viruses can also be applied for screening antiviral ISGs. In addition, RCREVs can be used for siRNAs HTS with high efficiency. For instance, a reporter CSFV expressing the Fluc gene has been used to screen antiviral siRNAs efficiently [16]. Recently, a recombinant EBOV carrying a luciferase reporter was used to screen siRNAs with higher screening efficiency than the wild-type virus [25].

However, there are some problems associated with RCREVs in HTS applications. First, the interference of compound fluorescence may occur when screening antivirals using fluorescent reporter-expressing viruses. Second, the antiviral effects of screened out antivirals by RCREVs need to be verified with the parental viruses. Furthermore, the antiviral effects may be different between RCREVs and the wild-type viruses due to the occasionally inclusive fluorescence signals for the wild-type viruses in indirect immunofluorescent assay (IFA) and higher sensitivity for RCREVs in Fluc/Rluc activity assay. Third, RCREVs are not ideal tools for screening of antivirals targeting specific step(s) of viral infection, since RCREVs can undergo a complete virus life cycle. For example, currently, a set of ISGs against hepatitis C virus (HCV), yellow fever virus (YFV), West Nile virus (WNV), Chikungunya virus (CHIKV), Venezuelan equine encephalitis virus (VEEV) and human immunodeficiency virus (HIV-1) have been documented, but their exact antiviral step(s) remain(s) unknown [35,36,37,38]. A practical challenge lies in the explanations of their antiviral mechanisms for antiviral ISGs screened by RCREVs. Despite these limitations, the following strategies may address some of the above issues. Fluc, Rluc and other bioluminescent reporters provide a viable alternative to fluorescent reporters in HTS assays for drug discovery [39]. This facilitates the development of highly sensitive, cell-based reporter assays [40], eliminates the problem of compound fluorescence [41], and possesses several advantages such as high reliability, convenience and adaptability to HTS assays. Remarkably, primary HTS followed by validation using traditional assays based on the parental viruses will greatly aid the discovery of novel antivirals against infectious diseases. Finally, the use of replicons or pseudoparticles would help to identify the step(s) of the viral life cycle as the potential targets of antivirals.

## 5. Applications of RCREVs in Basic Research

### 5.1. In Identification of Cellular Receptors/Membrane Proteins

Identification of cellular receptors facilitates understanding of the mechanisms of virus entry into host cells [42,43]. Moreover, the receptors are regarded as promising targets for development of novel antivirals [44,45,46,47]. While reporter-expressing pseudoparticles are widely used to screen viral receptors [48,49], RCREVs carrying Fluc [18,50], GFP [51] or Neo^R^ [52] as new useful tools have been applied for screening of viral receptors (Table 1). Since RCREVs can infect the cells with multiple life cycles in contrast to pseudoparticles, more false-positive receptors may be screened. In spite of these few limitations, RCREVs are still powerful tools to screen viral receptors in combination with unsusceptible cells and cDNA library derived from susceptible cells [51,52] or a set of siRNAs against a number of genes encoding cell membrane proteins [18,50] (Table 1).

### 5.2. Virus Tracking and Live Imaging in Vitro and in Vivo

With the development of reverse genetics systems, RCREVs provide an ideal tool for monitoring the dynamics of viral infection progression *in vitro* and *in vivo* due to eliminating the need for secondary labeling, which represents a significant advance in the study of the biology of viruses (Table 2).

RCREVs carrying a GFP reporter gene have been successfully used for tracking viral protein(s) or viral infection *in vitro* and *in vivo* [53,54,55,56], which indicates that the GFP reporter gene is suitable for generating RCREVs to track viral proteins either in cell cultures or animal models. Furthermore, GFPs in RCREVs can be expressed efficiently in rodent brain for a long time [57] and show lower autofluorescence in the tissue [56]. Therefore, GFP may be a promising option when RCREVs are used to study the infection of viruses replicating in the brain. Additionally, an engineered virus expressing the split-green fluorescent protein (split-GFP) in the presence of cell lines expressing the complementing GFP can facilitate the tracking of viral infection in living cells [58].

Compared with the most commonly used EGFP tag, the TC tag enables the fusion protein to fluoresce more quickly, with a minimum risk of disrupting the overall structure and function of the targeted protein [59]. The TC-labeling technology has led to successful tracking of the nonstructural or structural proteins of diverse viruses [60,61,62,63,64,65]. However, since the TC-tag technology contains a biarsenical labeling process [66,67], the engineered replication-competent TC-tagged viruses are not suitable for tracking viral protein *in vivo*.

In addition, recombinant canine distemper virus (CDV) expressing dTomato was used to investigate the routes of virus spread *in vivo* [56]. A fully functional recombinant pneumonia virus of mice (PVM) with Katushka 2 has been developed to track infection of target cells *in vivo* [68]. Compared with far-red GFP-like proteins, iRFP has a substantially higher signal-to-background ratio in a mouse model due to its infrared-shifted spectra [69,70]. Interestingly, the Cre recombinase as a reporter is used to generate RCREVs for visualizing virus infection in engineered cell lines or transgenic animals harboring a loxP-flanked fluorescent marker upstream of another otherwise silenced fluorescent reporter [71].

Recently, several influenza viruses expressing fluorescent proteins of different colors (“Color-flu” viruses expressing ECFP, EGFP, Venus or mCherry) or a toolbox of influenza A and B reporter viruses were generated to facilitate the study of viral infection in *in vivo* models. Whole-mount images of transparent lung tissues were obtained using a fluorescent stereomicroscope [72,73,74,75,76]. In addition, bioluminescent and fluorescent dual-reporter Marek’s disease viruses are engineered to track viral replication in cell cultures or animal models [77]. In the future, “color” or dual-reporter viruses will be powerful tools to analyze viral infection at the cellular level *in vivo* to better understand the pathogenesis of various viruses.

Notably, reporters fused with viral proteins are very suitable for investigating the localization and distribution of the proteins in infected living cells. RCREVs will help advance virus-related live-imaging studies *in vitro* and *in vivo*, which allow localization of the infection and tracking of changes in the distribution of viruses in animals in real time.

### 5.3. In Identification of Virus-Host Interactions

Elucidating various aspects of pathogen-host interactions is essential for the comprehensive understanding of pathogenesis. Compared with the most frequently used techniques for mapping of virus-host interactions, the approaches based on RCREVs can recapitulate the virus life cycle [78]. Split-Gluc (Gluc1 and Gluc2) has been applied for identification of virus-host interactions. For example, a recombinant influenza virus carrying a Gluc1-tagged polymerase subunit is used to infect the cultured cells expressing a pool of Gluc2-tagged cellular proteins involved in nucleocytoplasmic-transporting pathways for confirming virus-host interactions [79]. In addition, split-GFP reporter has huge potential in this application. However, the reporter activity based on the interactions of RCREVs with the cellular proteins may not be detected due to the interference of the space structure.

## 6. Other Applications

The RCREVs are also useful in modified live vaccines containing genetic markers, which have been developed for many viruses by inserting EGFP as a positive marker [80,81,82]. For example, a genetically marked recombinant rinderpest vaccine expressing GFP has been developed [81]. In addition, a recombinant GFP-tagged PRRSV containing a deletion of an immunogenic epitope, in accompany with the diagnostic tests (GFP- and epitope-based ELISAs), enables serological differentiation between the marker virus-infected animals and those infected with the wild-type virus [82]. A recombinant viral hemorrhagic septicemia virus (VHSV) harboring RFP gene was utilized to evaluate VHSV-based viral-vectored vaccines [83]. More recently, the marker vaccine vSMEGFP-HCLV3’UTR in the context of the CSFV Shimen strain was generated by inserting EGFP to create a positive marker [84].

For those viruses causing CPEs, RCREVs can be used as an intermediate to generate and purify expected variants. For example, a novel gE-deleted pseudorabies virus (PRV) was obtained by gE/gI-deleted virus expressing EGFP [85]. In addition, Katushka 2 as a reporter was used to evaluate a novel reverse genetic system of RSV [19].

Interestingly, oncolytic recombinant viruses harboring reporter genes have been developed and applied for the disease progression tracking and accurate visualization of tumor burden [14]. Since oncolytic viruses selectively infect as well as replicate within cancer cells, the recombinant oncolytic viruses expressing reporter genes, particularly for far-red fluorescent proteins, will be a promising option for real-time monitoring of viral infection in cancer tissues [14].

While RCREVs harboring a reporter fused to a viral protein are the most suitable for studying the localization of the protein in infected cells, RCREVs carrying separate reporters are useful for other basic research purposes. For example, the preferential translation of viral RNAs over host RNAs during VSV infection has been demonstrated by the EGFP reporter expressed from the recombinant VSV [86]. Recently, the contribution of EBOV proteins in modulating dendritic cells (DC) maturation was investigated using the recombinant virus carrying EGFP [87]. Furthermore, unique profiles of RFP expression acquired from thousands of co-infected cells with viable and defective viruses showed how the interference of defective viruses acts at multiple steps of infection [88].

## 7. Limitations and Prospects

Firstly, a practical challenge for some viruses lies in not allowing the insertion of reporter genes. As we stated above, it is difficult to insert a reporter gene into the genome of influenza viruses. Despite the challenge, reporter-expressing influenza viruses have been developed and applied in basic science [58,61,71,72,73,74,75,76]. To address the question, there are three necessary considerations, including the reporter protein itself, expression strategy, and structure of the viral protein. For example, the loop/linker regions are usually chosen to insert the TC tag based on the structure of NS1 of influenza viruses [61].

Although RCREVs have been developed and applied *in vitro* and *in vivo*, one question arises regarding the expression stability of the reporter gene in RCREVs during the viral replication *in vitro* and *in vivo* [53,89]. One potential consequence of RCREVs’ attenuation is the purging of the inserted reporter from the viral genome. In this regard, we need to better understand the mechanism of regulation of viral genome replication and gene expression [90,91], the association between structure and function of viral proteins, as well as the application of novel reporters such as NanoLuc due to its small size [92].

One of the biggest challenges is that RCREVs are possibly attenuated and may not accurately reflect natural infections [93,94], which partially limits the applications of the RCREVs, especially *in vivo*. Replacement of currently used expression strategies may be a promising approach to overcoming this problem. As an example, IRES or 2A peptide-encoding sequence has been used to express separately the reporter from viral protein [71,95]. Importantly, the use of split-GFP or split-luciferase may not compromise viral replication competency due to their smaller sizes [58,79]. However, whether these reporter viruses will be attenuated *in vivo* needs further investigation in the future. More recently, it has been reported that after mouse adaptation, influenza virus H5N1 expressing the Venus reporter gene became more pathogenic to mice and the Venus gene was more highly and stably expressed [96], which may be another promising avenue that maintains the pathogenicity of the reporter viruses.

Luciferase imaging uses the luciferases to catalyze reactions that produce visible light *in vivo* at body temperature, which is used to determine the sites of virus replication, monitor viral dissemination in real time [97]. However, there are many caveats in the process of obtaining accurate luciferase imaging [98]. For example, the reporter signal from RCREVs is attenuated when *in vivo* imaging in tissues. Despite these limitations, luciferases will still become major reporters for *in vivo* imaging in real time in the future as they have a number of advantages compared with the fluorescent reporters, such as no intrinsic autoluminescence. In addition, iRFPs are in high demand for *in vivo* imaging, which exhibit high brightness in mammalian cells and tissues and are suitable for long-term studies with multicolor imaging.

Finally, in view of the advantages and disadvantages of different reporters, there seems no universal reporter for various applications. Fortunately, ever-increasing novel reporters, including GFP mutants, “red-shifted” analogs of luciferase, variants of luciferase and novel luciferase NanoLuc, can be chosen to design RCREVs for specific purposes. Moreover, the dual-reporter RCREVs may be widely used to address the scientific questions. Although reporter-based assays require costly automated imaging equipment, the detection of the reporter gene expression could be also performed with inexpensive, small and simple-to-use equipment, such as a PCR device based on the development of the technologies discussed in this article.

## 8. Conclusions

RCREVs have proved themselves to be powerful tools for applied and basic sciences. Despite their limitations, RCREVs have many more far-reaching benefits in virus research: a genome-wide RNAi screening for host factors required for virus replication, identifying antivirals against viral infections using HTS settings, monitoring viral infections *in vitro* and *in vivo* in real time, or evaluating vaccination approaches, as well as detecting antiviral NAbs.

## Figures and Tables

**Table 1:** Cellular receptors screened by representative RCREVs.

Reporters	Viruses	Expression Strategies	Screened Cellular Receptors Proteins
Firefly luciferase (Fluc)	Classical swine fever virus (CSFV)	Fusion with a viral protein	Laminin receptor (LamR) [50]
	Alphaviruses	Introduction of foot-and-mouth disease virus 2A-enconding sequence	Fuzzy homolog (FUZ) and tetraspanin membrane protein TSPAN9 [18]
Green fluorescent protein (GFP)	Porcine reproductive and respiratory syndrome virus (PRRSV)	Fusion with a viral protein	CD163 [51]
Neomycin resistance gene (Neo^R^)	Equine infectious anemia virus (EIAV)	Introduction of an additional transcriptional unit	Equine lentivirus receptor 1 (ELR1) [52]

**Table 2:** Applications of representative RCREVs in virus tracking and live imaging *in vitro* and *in vivo*.

Reporters	Viruses	Tracking and Live Imaging
Green fluorescent protein (GFP)	Influenza virus	Dynamics of virus infection progression in mice [53]
	Herpes simplex virus (HSV)	Compartmentalization of protein by autofluorescent particles [54]
	Borna disease virus (BDV)	In rodent brains [57]
	Canine distemper virus (CDV)	Routes of virus spread *in vivo* [56]
	Vesicular stomatitis virus (VSV)	Intracellular transport [55]
Tetracysteine (TC)	Vesicular stomatitis virus (VSV)	Dynamic imaging of M protein and virus uncoating in infected cells [60]
	Influenza A virus	Visualization of NS1 protein nuclear import in virus-infected cells in real time [61]
	Classical swine fever virus (CSFV)	Nucleus import and export [62]
	Hepatitis C virus (HCV)	Virus particle assembly [63]
	Human immunodeficiency virus (HIV)	Viral component complexes [64] *de novo* HIV production [65]
ECFP, EGFP, Venus, RFP, mCherry, NanoLuc and Gluc split-GFP, Cre recombinase	Influenza A/B virus	Viral infection *in vitro* or in lung tissues [58,71,72,73,74,75,76]
Katushka 2	Pneumonia virus of mice (PVM)	Tracking of viral infection of target cells *in vivo* [68]
iRFPs	Adenovirus	In mouse model [69]
dTomato	Canine distemper virus (CDV)	Routes of virus spread *in vivo* [56]
EGFP+Rluc/Gluc	Marek’s disease virus (MDV)	Tracking of viral replication *in vitro* and *in vivo* [77]
